# Matrix metalloproteinases and epileptogenesis

**DOI:** 10.1186/s40348-014-0006-y

**Published:** 2014-11-04

**Authors:** Chrysanthy Ikonomidou

**Affiliations:** Department of Neurology, University of Wisconsin, Madison, WI 53705 USA

**Keywords:** Epilepsy, Extracellular matrix, Prevention, Synaptic plasticity, Neuroinflammation, Cell death

## Abstract

Matrix metalloproteinases are vital drivers of synaptic remodeling in health and disease. It is suggested that at early stages of epileptogenesis, inhibition of matrix metalloproteinases may help ameliorate cell death, aberrant network rewiring, and neuroinflammation and prevent development of epilepsy.

## Introduction

Epilepsy is one of the world's oldest recognized disorders, affecting around 50 million people worldwide. Despite continuing advances in treatment options, 30% of patients remain drug refractory. Annual costs associated with epilepsy are immense; in Europe alone, they amount to over €20 billion. In addition to the costs, the social burden and increased risk of death associated with the disease underline the urgent need to find ways to prevent epilepsy.

The development of chronic epilepsy can be the result of initial insults (e.g., tumor, infection, stroke, traumatic brain injury), brain malformations (e.g., focal cortical dysplasias, schizencephaly, and others), mutated genes (genetic epilepsies), or a component of a neurodevelopmental or neurodegenerative disorder (e.g., fragile X syndrome, Rett syndrome, neuronal ceroid lipofuscinosis). Often, there is a time lag between the first impact of the triggering factor and the occurrence of the first spontaneous seizure. During this period, the brain is functionally altered and primed to generate abnormal electrical activity. This process, named epileptogenesis, can take several years in humans and a few weeks in experimental animal models to produce epilepsy [1]. Extensive network modifications occur during epileptogenesis; they are believed to play a key role in the construction of epileptogenic circuits. Some of these alterations are a direct consequence of the initial trigger; others evolve during epileptogenesis. Cell death, aberrant synaptogenesis, and synaptic plasticity and neuroinflammation constitute three key mechanisms implicated in epileptogenesis.

Classical anticonvulsants are ineffective in preventing the process of epileptogenesis. To date, there are no effective antiepileptogenic treatments [1].

### The role of the extracellular matrix in epileptogenesis

The extracellular matrix (ECM) occupies the space between neurons and glial cells and regulates neuronal cell development, activity, and growth. It contributes to the structural stabilization of neuronal processes and synaptic contacts during the maturation of the central nervous system. The remodeling of the ECM during development and after central nervous system injuries affects neuronal guidance, synaptic plasticity, and regeneration (see review by Soleman et al. [2]).

Neurons and glial cells secrete diverse molecules that contribute to the composition of the ECM. The quantity of ECM relative to cell mass is very high during embryonic development and declines towards the time of birth, while the composition of matrix proteins also changes. The adult ECM restricts major reorganization of processes and axonal outgrowth through the differential expression of molecules into adulthood, and the appearance of cartilage-like structures called perineuronal nets. Nevertheless, the adult central nervous system (CNS) still retains a capacity to promote structural plasticity in response to injury and blood-brain barrier disruption and matrix metalloproteinases (MMPs) play key roles in that respect (see review by Soleman et al. [2]).

### Matrix metalloproteinases

A family of extracellular soluble or membrane bound neutral proteases, cleave, and remodel the ECM (review by Yong [3] and Rosenberg [4]). Their substrates include proteinases, growth factors, cytokines, cell surface receptors, and cell adhesion molecules (Table 1). Cleavages of these substrates by MMPs have been implicated in the regulation of diverse biological processes under normal and pathological conditions, including embryonic development, wound repair, inflammatory and neurological diseases, and cancer as well as spinal cord and brain injuries. MMP expression and enzymatic activity markedly increase in response to brain injury in the developing and the adult brain [3].

**Table 1:** **MMP substrates in synapses**

**Target**	**MMP**	**Receptors/binding partners**	**Synaptic effect**
ECM proteins			
Laminin	MMP2,3,7,9,11,12,19,25	Integrins	Synapse formation and remodeling, NMDA-R activity, hippocampal LTP
Brevican	MMP1,2,3,7,8,10,13	Tenascin-R, hyaluronan	Hippocampal LTP
Tenascin-R	MMP14,15	Brevican	Synapse formation, hippocampal LTP, learning and memory
	MMP1,2,3,7,8,13		
Growth factors			
TNF-α	MMP14,15,16	TNFR1, TNFR2	AMPA receptor trafficking, synaptic scaling
	MMP1,2,3,7,8,9,12,13		
BDNF	MMP2,3,7,9	trkB, p75	Hippocampal LTP, learning and memory
ProBDNF	MMP9		
ProNGF	MMP7	p75	Cell survival
Membrane proteins			
N-Cadherin	MMP24	N-Cadherin, catenin	Synapse formation and stability, LTP
β-Dystroglycan	MMPs	α-Dystroglycan, dystrophin	Hippocampal LTP
Ephrin-B2	MMPs	EphB receptors	Synapse formation and stability, hippocampal LTP, learning and memory

Adapted and modified from Ethel and Ethel [5].

**Figure 1:**
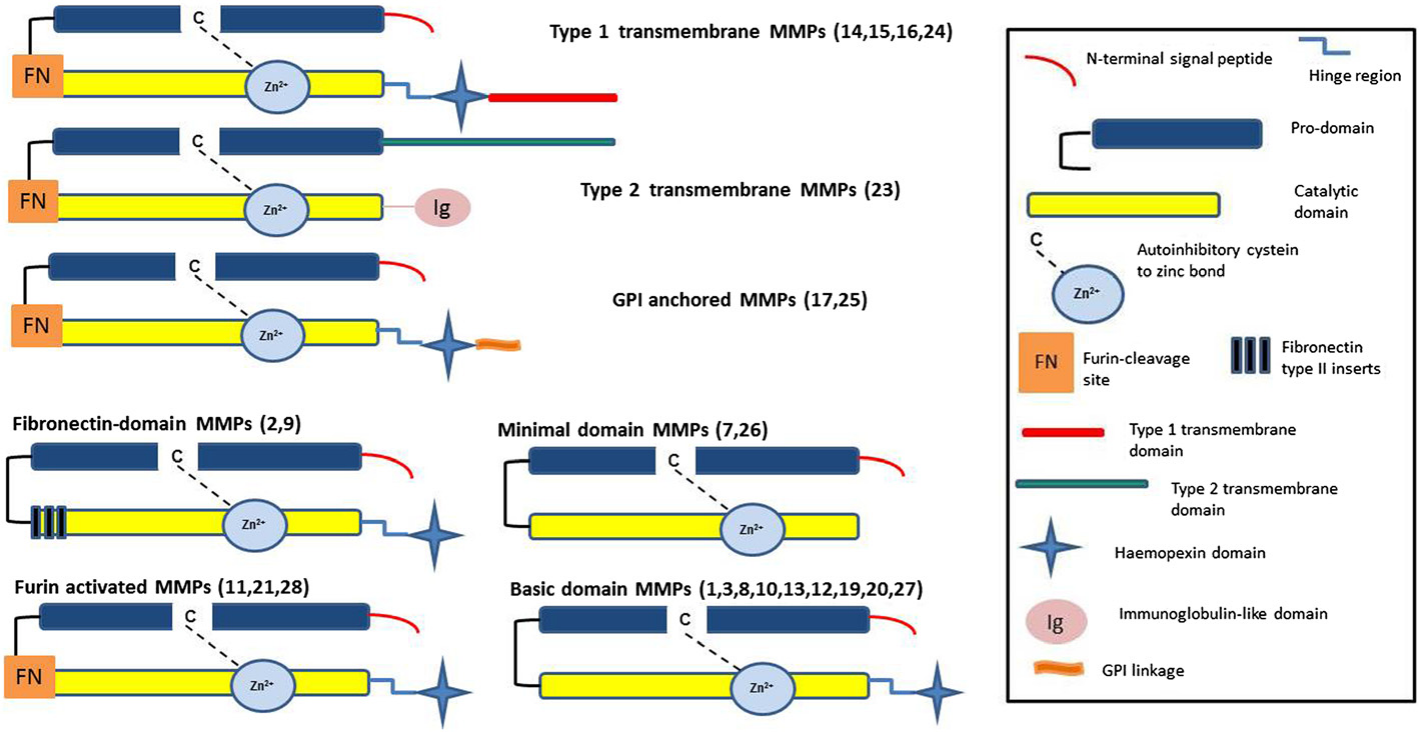
**Structure of matrix metalloproteinases (adopted and modified from Huntley [** 6 **]).**

MMPs are multidomain proteins named according to a sequential numbering scheme (Figure 1). In humans, 24 MMP genes encode 23 distinct MMPs (two identical genes located on chromosome 1 encode MMP23). MMPs all possess an N-terminal signal peptide, an autoinhibitory pro-domain, and a catalytic domain. Most MMPs also possess a C-terminal haemopexin domain which contributes to the target specificity of MMP proteolysis by coordinating interactions with substrates. The haemopexin domain mediates protein-protein interactions and can anchor MMPs to other cell-surface proteins. MMPs can also act as ligands through their haemopexin domain by binding to receptors (see review by Huntley [6] and Rosenberg [4]).

The majority of MMPs are secreted into the ECM, while seven MMPs are membrane-associated, anchored by a type 1 or type 2 transmembrane domain or a glycosylphosphatidylinositol (GPI) linkage.

MMPs are synthesized as inactive zymogens and activated by disruption of the cysteine-zinc bond through other MMPs and serine proteases or via modification of the thiol-group (oxidation or *S*-nitrosylation [7]). Although MMP activity is mostly extracellular, some active MMPs can be found in neuronal or glial nuclei in which they may have transcription factor-like or DNA repair-like activity. MMP activity is terminated in the brain mostly by four secreted tissue inhibitors of metalloproteinases (TIMPs) (see review by Huntley [6]).

Higher MMP expression and activity in the developing brain and MMP knockout studies reporting CNS defects suggest that MMPs play crucial roles in neural development. MMPs have been shown to control brain function by regulating neurogenesis, cell migration, topographic mapping, axon guidance, myelination, and synaptogenesis. More recently, MMPs have been suggested as regulators of synaptic activity in hippocampus, learning, and memory (review by Huntley [6]).

Among MMPs, MMP-2 and matrix metalloproteinase-9 (MMP-9) are most abundantly expressed in the brain. MMP-9 is highly expressed in neuronal cell bodies and dendrites. Growing data suggest the association of MMP-9 with dendritic spine remodeling, synaptic plasticity, and learning and memory formation. Experimental evidence further suggests that MMPs are involved in seizure-induced cell death, breakdown of the blood-brain barrier, neuroinflammation, and aberrant synaptic plasticity, all of which occur in the context of epileptogenesis [3,4,8,9].

### Actions of MMPs in the brain with potential relevance to epileptogenesis

#### MMPs and cell death

Apart from the proteolysis of extracellular protein targets, MMPs also localize to various intracellular sites and have intracellular actions which consist of them rapidly acting on intracellular substrates in response to brain injury during hypoxia, traumatic injury, and prolonged seizure activity. Increased activity of MMPs including MMP-2, MMP-9, and MMP-13 was demonstrated in neuronal nuclei of brains at an early stage of ischemic stroke and reperfusion injury [4,10]. The nucleus has a matrix that resembles the ECM and provides structural and organizational support for various nuclear processes as well as apoptosis, which involves proteolytic processing of nuclear proteins. The intranuclear gelatinase activity in ischemic neurons suggests a possible role for gelatinases in nuclear matrix proteolysis. Intranuclear MMP-2 and MMP-9 activity was shown to facilitate oxidative injury in neurons during early ischemic insult by cleaving nuclear proteins poly-ADP-ribose polymerase 1 (PARP-1) and X-ray cross-complementary factor 1 (XRCC1). Both PARP-1 and XRCC1 are DNA repair enzymes which become activated following the induction of DNA damage. DNA base excision repair machinery in neuronal nuclei repairs oxidative DNA damage. DNA strand breaks induce PARP-1 activity, which triggers DNA repair by binding to damaged DNA. The cleavage of DNA repair proteins by MMP activity in neuronal nuclei interferes with oxidative DNA damage repair, which could contribute to neuronal apoptosis. Mice overexpressing superoxide dismutase (SOD) are protected from ischemic injury and have reduced MMP production.

Activation of intracellular MMPs could be one of the earliest pathological events triggered downstream of oxidative stress. MMP inhibition may prove to be a novel therapeutic strategy to prevent neuronal apoptotic injury in the brain. A crucial role of MMPs in mediating neuronal death in the developing mammalian brain has been demonstrated in animal models for traumatic, ischemic, and epileptic brain damage [11,12].

#### MMPs and synaptic plasticity

MMP-9 is required for late-phase long-term potentiation (LTP) in the hippocampus [13] and prefrontal cortex [14] and for spatial and emotional learning [13]. Bozdagi et al. [15] and Tian et al. [16] demonstrated that LTP is associated with significant increases in levels of MMP-9, this effect being dependent upon *N*-methyl-d-aspartate (NMDA) receptor activation. MMP-9 appears to exert its action directly at the level of dendritic spines [8,16,17], possibly via cleavage of synapse-associated molecules such as dystroglycan [8,10] and intracellular adhesion molecule 5 (ICAM-5) [16].

Transsynaptic activation of NMDA receptors in neurons leads to activation of the ERK1/2 and PI3 kinase pathways [18]. The hypothesis has been formulated that, in the context of evolving epilepsy, pathological transsynaptic activation of NMDA receptors leads to increased MMP-9 transcription via ERK1/2 and/or PI3 kinase-dependent mechanisms [8]. MMP-9 sequentially remodels dendritic spines within secondary epileptogenic foci in the neocortex and eventually the hippocampus, partly via ICAM-5 and dystroglycan cleavage. MMP-9 may exert its effects on integrins through the cleavage of laminin or other ECM components, exposing otherwise inaccessible RGD sites that can then induce integrin signaling. Synapse remodeling induced by MMPs can be blocked by the NMDA receptor antagonist MK-801, indicating that MMPs' effects are mediated through NMDA receptor activity. In fact, NMDA receptor activity has been reported to regulate MMP-9 protein levels and activity, which may be a key step in LTP, the neurophysiological correlate of learning and memory. MMP-9 knockout mice show behavioral impairments in hippocampus-dependent associative learning [13]. Furthermore, hippocampal slice cultures from MMP-9 knockout mice show impaired LTP, which can be restored by the application of recombinant MMP-9 [13].

MMP-7 has also been shown to modulate synapse morphology. Active MMP-7 alters the morphology of dendritic spines, inducing their elongation and remodeling. MMP-7 is derived from macrophages and is present at sites of tissue repair and remodeling. MMP-7 can process pro-MMP-9 into the fully active form and may exert its effects on synapses through activation of neuron-derived MMP-9.

MMP-24 is another potential regulator of synapse formation and remodeling, as it is developmentally regulated in the cerebellum and hippocampus and is also found in the adult brain. It is expressed in neurons and may regulate axon growth and dendrite extension. MMP-24 interacts with synaptic scaffold proteins, AMPA receptor binding protein (ABP) and glutamate receptor interacting protein (GRIP) at synaptic sites. Because of the furin cleavage sites, MMP-24 appears on the cell surface in an active form and may thus affect synapse remodeling by cleaving cell adhesion molecules within and around CNS synapses (reviewed by Huntley [6]).

#### MMPS and neuroinflammation

The blood-brain barrier (BBB), a unique feature of the cerebral vasculature, is gaining attention as a feature in common neurologic disorders including epilepsy. Although acute blood-brain barrier dysfunction can induce cerebral edema, seizures, or neuropsychiatric symptoms, epileptogenesis and cognitive decline are among the chronic effects (reviewed by Rosenberg [10] and Schoknecht and Shalev [19]).

BBB dysfunction acutely promotes single seizures, facilitates the development of epilepsy in animal models, and furthermore was observed in patients with mesial temporal lobe epilepsy. Signaling cascades modulating BBB properties may become future targets to prevent seizures and epileptogenesis. Blocking transforming growth factor β (TGF-β) receptors reduced albumin-mediated epileptogenesis, suggesting TGF-β signaling to mediate downstream events following focal neocortical BBB opening that led to albumin extravasation and epileptogenesis. Microarray analysis further revealed that focal BBB opening and topical application of albumin and TGF-β similarly induced astrocytic activation, downregulated genes related to γ-aminobutyric acid (GABA) ergic signaling, and led to an inflammatory response. Astrocytic activation leads to altered extracellular homeostasis including impaired potassium buffering and glutamate metabolism, consequently increasing neuronal excitability. Inflammatory mediators such as tumor necrosis factor α (TNF-α), interleukin (Il)-6, and IL-1β have promoted seizures and epileptogenesis. In preliminary studies, patients have responded to antiinflammatory treatment with reduced seizure frequency, thus highlighting the immune response as a target for therapeutic interventions (reviewed by Rosenberg [10] and Schoknecht and Shalev [19]).

There is ample evidence to suggest that MMPs are key players in enhancing BBB permeability in the context of brain insults and, by doing so, promoting neuroinflammation. The basal lamina around cerebral blood vessels contains extracellular matrix proteins, including laminin, fibronectin, heparan sulfate, and type IV collagen. Proteolysis of the blood-brain barrier by MMPs results in loss of basal lamina proteins. MMP-2, MMP-3 and MMP-9 increase the permeability of the blood-brain barrier, and inhibitors of MMPs can reduce damage to the blood-brain barrier. In an *Mmp9* knockout model, focal ischemic lesions decreased the damage to the blood-brain barrier and the infarct size.

MMP3 is an inducible enzyme and its concentration increases in hypoxia-ischemia and immunological reactions. When *Mmp3* is knocked out, the normal disruption of the blood-brain barrier that occurs after intracerebral injection of lipopolysaccharide is attenuated. *Mmp3* knockout mice have fewer neutrophils recruited to the site of inflammation than do wild-type mice (reviewed by Rosenberg [10] and Schoknecht and Shalev [19]).

### The role of MMPs in animal models of epileptogenesis

Recent data indicate an involvement of extracellular proteolysis in the pathogenesis of epilepsy. Activation of MMP-2 and MMP-9 occurs in the brain in the kainic acid rat seizure model. Particularly intriguing is the evidence for a role of the matrix metalloproteinase-9 (MMP-9). Several studies have shown robust activation of MMP-9 by seizure-evoking stimuli. The first crucial study linking MMP-9 to epileptogenesis was published by Wilczynski et al. [8]. MMP-9 was found to promote epileptogenesis in kainate-evoked and pentylenetetrazole-kindling-induced epilepsy in rats and mice. In two animal models of temporal lobe epilepsy, the kainic acid model and the pentylenetetrazole kindling model, these authors demonstrated decreased sensitivity in MMP-9 knockout mice but increased sensitivity in transgenic rats overexpressing MMP-9. By means of immuno-electron microscopy, it was shown that MMP-9 associates with hippocampal dendritic spines bearing asymmetrical (excitatory) synapses. Both the MMP-9 protein levels and enzymatic activity strongly increase upon seizures. MMP-9 deficiency in MMP-9 knockout mice diminished seizure-evoked pruning of dendritic spines and decreased aberrant synaptogenesis and mossy fiber sprouting. The observations that aberrant synaptic plasticity contributes to epileptogenesis and that MMP-9 is a key molecule for synaptic plasticity acting via β1 integrins suggest that MMP-9 could play a crucial role in epileptogenesis through a similar mechanism (Huntley [6]).

In humans, high serum levels of MMP-9 were detected in children following febrile seizures. Prolonged seizures were associated with high serum MMP-9 levels and increases in the ratio of MMP-9 to TIMP-1 in patients with acute encephalopathy with dysfunction of the blood-brain barrier following prolonged febrile seizures [20]. MMP-9 protein levels were elevated in cortical lesions in patients with focal cortical dysplasia type IIb and tuberous sclerosis complex, which cause chronic epilepsy in children, suggesting a possible pathological role for MMP-9 in these intractable conditions. Another study showed that the MMP-9 levels in cerebrospinal fluid were higher in patients with bacterial meningitis who developed secondary epilepsy than in individuals who recovered without neurological deficits, suggesting that MMP-9 concentrations contribute to postmeningitic neurological sequelae (reviewed by Mizoguchi and Yamada [21]).

## Summary and conclusions

To date, there is no armamentarium available to prevent the development of epilepsy. Antiepileptic treatments do not influence epileptogenesis. The ECM is gaining increasing attention as a compartment in which remodeling programs primarily destined to operate during development are reactivated following injurious insults and contribute to aberrant rewiring of neuronal networks that results in pathologically increased excitability and the development of epilepsy.

In this minireview, evidence for the role of a class of enzymes, the MMPs, in mediation of cell death, aberrant synaptic plasticity, and neuroinflammation in the mammalian brain is presented briefly with appropriate reference to more extensive reviews.

The suggestion is presented that at early stages of epileptogenesis, it could be beneficial to prevent neural network rewiring and resulting ECM remodeling via the inhibition of MMPs. Since MMPs and other ECM ectoproteases also play crucial roles in neurologic recovery, the major challenge will be to direct reactivated structural plasticity in the ‘right’ direction. For that, it will be critical to determine how MMP activity transitions from its normal role in synaptic circuit remodeling to its aberrant and deleterious roles that lead to epilepsy.

A large number of MMP inhibitors have been developed in the past 30 years for the treatment of metastatic cancer, and several generations of synthetic MMP inhibitors were tested in clinical trials since the 1990s [22]. These include the first-generation peptidomimetics (such as Batimastat and Marimastat), the second-generation nonpeptidomimetics (such as Tanomastat and Prinomastat), and the third-generation tetracycline derivatives (such as Minocycline and Metastat). Despite encouraging preclinical data in cancer, clinical trials were unsuccessful mainly because of the lack of overall response and the presence of dose-limiting toxicity. Consequently, all clinical trials on the use of synthetic MMPIs in cancer have been terminated [23]. The information presented in this minireview suggests a potential new field of application for these agents. Testing of available MMP inhibitors in preclinical models of epileptogenesis has not been pursued so far and seems to be the next most logical step to determine whether MMP inhibition may constitute a promising antiepileptogenic strategy.
